# Fish oil supplementation enhanced CPT-11 (irinotecan) efficacy against MCF7 breast carcinoma xenografts and ameliorated intestinal side-effects

**DOI:** 10.1038/sj.bjc.6690713

**Published:** 1999-10

**Authors:** W E Hardman, M P Moyer, I L Cameron

**Affiliations:** Departments of Cellular and Structural Biology, Surgery, The University of Texas Health Science Center at San Antonio, San Antonio, TX 78284, USA; INCELL Corporation, LLC, 12 000 Network Boulevard, Suite B-200, San Antonio, TX, 78249, USA

**Keywords:** fish oil, breast cancer, CPT-11, irinotecan, intestinal side-effects

## Abstract

The cancer chemotherapeutic efficacy of the topoisomerase I inhibitor, CPT-11 (irinotecan) is often limited by the induction of severe delayed diarrhoea. In animal studies, CPT-11 use is associated with histopathological damage to the mucosa of the small and large intestines. Results from the present study demonstrate that 60 mg CPT-11 per kg body weight (i.v. q4d × 6) halted the growth, but did not cause significant regression, of MCF7 human breast carcinoma xenografts in mice fed a diet containing 7% corn oil. However, when the diet of the MCF7-bearing mice was supplemented with 3% or 6% fish oil, the same CPT-11 treatment caused significant regression of the MCF7 xenograft. Histomorphometric analyses of intestinal mucosa of mice treated with CPT-11 and fed the diet containing 7% corn oil indicated that treatment with CPT-11 induced structural changes in the intestinal mucosa which persisted at least 5 days after the last dose of CPT-11. The intestinal mucosal architecture of mice that were treated with CPT-11 and fed the diets containing fish oil was largely unchanged from the architecture of the group of mice which did not receive CPT-11. These findings indicate that fish oil supplements may be a useful adjunct to CPT-11 treatment. © 1999 Cancer Research Campaign


*British Journal of Cancer* (1999) **81**(3), 440–448
© 1999 Cancer Research Campaign
Article no. bjoc.1999.0713

### Fish oil supplementation enhanced CPT-11 (irinotecan) efficacy against MCF7 breast carcinoma xenografts and ameliorated intestinal side-effects

**WE Hardman[1], MP Moyer[2,3] and IL Cameron[1]**

Departments of [1]Cellular and Structural Biology and [2]Surgery, The University of Texas Health Science Center at San Antonio, 7703 Floyd Curl Drive, San Antonio, TX 78284, USA; [3]INCELL Corporation, LLC, 12 000 Network Boulevard, Suite B-200, San Antonio, TX 78249, USA

**Summary** The cancer chemotherapeutic efficacy of the topoisomerase I inhibitor, CPT-11 (irinotecan) is often limited by the induction of severe delayed diarrhoea. In animal studies, CPT-11 use is associated with histopathological damage to the mucosa of the small and large intestines. Results from the present study demonstrate that 60 mg CPT-11 per kg body weight (i.v. q4d $\times$ 6) halted the growth, but did not cause significant regression, of MCF7 human breast carcinoma xenografts in mice fed a diet containing 7% corn oil. However, when the diet of the MCF7-bearing mice was supplemented with 3% or 6% fish oil, the same CPT-11 treatment caused significant regression of the MCF7 xenograft. Histomorphometric analyses of intestinal mucosa of mice treated with CPT-11 and fed the diet containing 7% corn oil indicated that treatment with CPT-11 induced structural changes in the intestinal mucosa which persisted at least 5 days after the last dose of CPT-11. The intestinal mucosal architecture of mice that were treated with CPT-11 and fed the diets containing fish oil was largely unchanged from the architecture of the group of mice which did not receive CPT-11. These findings indicate that fish oil supplements may be a useful adjunct to CPT-11 treatment. © 1999 Cancer Research Campaign

**Keywords:** fish oil; breast cancer; CPT-11; irinotecan; intestinal side-effects

CPT-11 (irinotecan hydrochloride, 7-ethyl-10-(4-[1-piperidino]-1-piperidino)-carboxyloxy-camptothecin) is a water-soluble derivative of camptothecin (Sawada et al, 1991). CPT-11 stabilizes the nuclear topoisomerase I/DNA complex leading to an accumulation of single-strand breaks in DNA and to cell death (Kawato et al, 1991; Rivory, 1996*a*; Takasuna et al, 1996). Enhanced lipid peroxidation may also contribute to CPT-11-induced cell death (Sadzuka and Hirota, 1997). Clinically, CPT-11 has been used to treat a number of cancer types including colorectal, breast, cervical, ovarian, gliomas, lymphoma and small-cell and non-small-cell lung (Rothenberg, 1996; Gerrits et al, 1997; Colvin et al, 1998). CPT-11 was found to be the most effective chemotherapeutic agent in our recent comparison of the efficacies of doxorubicin, cisplatin, CPT-11 and topotecan against xenografts of colon (SW 620, COLO 205 and HT29), lung (H 460, A549, H 226) and breast (MDA-MB 231, MCF7 and T47D) cancer cell lines in nude mice (manuscript in preparation).

In a previous study (Hardman et al, 1997), we used 19% (w/w) dietary fish oil to increase the efficacy of edelfosine, an ether-lipid cancer chemotherapeutic drug. One of the proposed mechanisms of fish oil's ability to increase the efficacy of edelfosine was by enhancing lipid peroxidation in the tumour to cytotoxic levels (Hardman et al, 1997). Since CPT-11 treatment has also been reported to increase lipid peroxidation (Sadzuka and Hirota, 1997), we questioned whether the addition of fish oil to the diet of cancer bearing mice prior to treatment with CPT-11 would increase the efficacy of CPT-11 against the tumour. To increase the clinical relevance of the current study, fish oil was incorporated at 3% or 6% w/w of the diet, so that the quantity of fish oil fed to the mice would approximate levels which humans can consume.

An important consideration prior to recommendation of any drug treatment is the evaluation of toxic side-effects to the host. The major dose-limiting toxicity of CPT-11 in humans has been a debilitating, delayed diarrhoea (Rivory, 1996*a*; Rothenberg, 1996; Kase et al, 1997*b*; Sakai et al, 1997). Histopathological changes in the small and large intestines, including degeneration and necrosis of villi and crypt cells (Ikuno et al, 1995; Kase et al, 1997*b*; Cao et al, 1998; Shinohara et al, 1998) have been associated with the diarrhoea resulting from CPT-11 administration in preclinical animal studies (Cao et al, 1998). Strategies that reduce the histopathological damage to the small and to the large intestine might be expected to increase the clinical usefulness of CPT-11.

In the current report, tumour growth curves revealed that supplementation of the diet with either 3% or 6% w/w fish oil fed prior to and during treatment with CPT-11 enhanced regression of the MCF7 human breast cancer xenografts in nude mice. Evaluation of side-effects of CPT-11 treatment showed that CPT-11 treatment caused the same body weight loss whether the mice were fed the corn oil or the fish oil diets; however, histopathological damage to the small and large intestine was reduced when the CPT-11 treated mice consumed dietary fish oil. Thus, consumption of low levels of dietary fish oil increased the efficacy of CPT-11 against the tumour and decreased the histopathological damage to the intestines caused by CPT-11 treatment. In this preclinical study, supplementation of the diet with low levels of fish oil proved to be an effective adjunct to chemotherapy with CPT-11 and may also be an effective adjunct to cancer chemotherapy with CPT-11 in humans.

*Received 23 November 1998*
*Revised 11 March 1999*
*Accepted 17 March 1999*

*Correspondence to:* WE Hardman

### METHODS AND MATERIALS

### Tumour cells

MCF7 human breast cancer cells (American Type Culture Collection, Rockville, MD, USA) were cultured for injection in nude mice. The culture medium was M3:10™ (INCELL Corporation, LLC).

### Mice

Twenty female nude mice (*nu/nu*, Harlan Sprague Dawley, Madison, WI, USA) 6 weeks old were used in this study. Each mouse was numbered for identification. The mice were allowed to acclimate for 1 week before beginning the experiment. Mice were housed under aseptic conditions (positive air pressure in a designated nude mouse room, cages, bedding and water cages and microisolator tops) in a temperature (24°C) and light controlled (12 h light per day) room. All mouse handling was carried out under a laminar flow hood. All animal use and handling was approved by the UTHSCSA Institutional Animal Care and Use Committee before commencing the experiment. The animal care facilities are accredited by the American Association for the Accreditation of Laboratory Animal Care.

### Oestrogen supplementation

Female nude mice produce inadequate oestrogen to support the growth of MCF7 cells. Therefore, the mice were given injections of β-oestradiol (Sigma, St Louis, MO, USA) dissolved in pure sesame oil (0.1 mg per 0.05 ml sesame oil per mouse, subcutaneously (s.c.) over rump) beginning 1 day before the injections of MCF7 cells and at weekly intervals thereafter until the end of the experiment.

### Preparation of cells

Cultured MCF7 cells were harvested, rinsed then suspended in serum-free M3D base culture medium (INCELL Corporation, LLC). Cells in suspension were counted using a haemocytometer and the cell count was adjusted to $10^8$ $ml^{-1}$. The suspension was kept well mixed during the time of injection. MCF7 cells ($5 \times 10^6$ cells in 0.05 ml of serum-free media) were injected s.c. on the upper back of each mouse.

### Experimental design

Mice were fed Harlan Teklad LM-485 Mouse Chow diet while the tumours were allowed to grow to about 5 mm diameter. This allowed the tumours to become established as growing tumours in the host mice before feeding of the experimental diets. The tumour-bearing mice were then divided into four groups. One group of five mice remained on the chow diet and did not receive CPT-11 (untreated, normal control). Three groups were placed on diets, based on the AIN-76A diet, modified to contain 7% total fat.

The 7% total fat for each of the three experimental diets was divided as follows:

1. 7% corn oil, 0% fish oil (control diet with CPT-11)
2. 4% corn oil, 3% antioxidant-free fish oil (3% fish oil with CPT-11)
3. 1% corn oil, 6% antioxidant-free fish oil (6% fish oil with CPT-11).

**Table 1** Composition of the experimental diets by weight per cent (g per 100 g of food)

| Ingredient | g per 100 g of food |
|---|---|
| [a]Total oil | 7.0 |
| Sugar | 48 |
| Casein | 20 |
| Cornstarch | 15 |
| AIN-76 vitamin mix[a] | 1.0 |
| AIN-76 mineral mix[a] | 3.5 |
| Choline bitartrate | 0.2 |
| DL-methionine | 0.3 |
| Cellulose | 5 |
| Total | 100.0 |
| Composition of the diets by % calories[b] | |
| Protein | 20.2 |
| Carbohydrate[c] | 63.9 |
| Fat | 15.9 |
| [b]Energy content of each diet, kcal/g | 3.95 |

[a]Total fat was 7%. Diet types included 7% corn oil (control diet with CPT-11) or 4% corn oil, 3% antioxidant-free menhaden fish oil (3% fish oil diet) or 1% corn oil, 6% antioxidant-free menhaden fish oil (6% fish oil diet). [b]Caloric content is calculated at 4 kcal/g for protein and carbohydrate and 9 kcal/g for fat. [c]The % of calories from carbohydrate include the calories from sucrose, cornstarch and sucrose in the vitamin and mineral mix. Diet components and chemicals – purified high nitrogen casein, pure corn starch, Alphacel (non-nutritive bulk cellulose) AIN-76 vitamin mixture, AIN-76 mineral mixture and choline bitartrate (99% pure) were obtained from ICN Nutritional Biochemicals, Cleveland, Ohio. Imperial brand (Sugarland, TX) extra fine pure cane sugar and 100% pure corn oil (Wesson) were purchased locally. DL-methionine (cell culture, Mr 149.2), antioxidant free menhaden fish oil and ferric citrate were purchased from Sigma, St. Louis, Missouri.

The compositions of the experimental diets are shown in Table 1. Fish oil was purchased and used without added antioxidant specifically to exploit the potential to enhance lipid peroxidation, as in our previous study (Hardman et al, 1997). The corn oil and/or fish oil diets were prepared weekly, daily portions for each cage were packaged individually and the packages were stored in sealed containers at –20°C to suppress spontaneous lipid peroxidation. The corn oil and the fish oil diets were replaced daily. The mice were maintained on these diets for 10 days before beginning treatment with CPT-11.

### Drug preparation

CPT-11 was obtained as irinotecan hydrochloride (Pharmacia & Upjohn, Kalamazoo, MI, USA). It was prepared, according to manufacturer's directions, to duplicate the clinical formulation (Camptosar™). The prepared CPT-11 contained: 20 mg $ml^{-1}$ irinotecan hydrochloride, 45 mg $ml^{-1}$ sorbitol (Sigma, St Louis, MO, USA) and 0.9 mg $ml^{-1}$ lactic acid (Sigma, St Louis, MO, USA) and was pH adjusted to 3–3.8. The solution was warmed in a 100°C water bath to dissolve the CPT-11. A dose of 60 mg CPT-11 per kg body weight (about 0.08 ml per 28 g mouse) was injected into the lateral tail vein of each treated mouse, once each 4 days for 6 weeks.

### Tumour and body weight measurements

Lengths and widths of tumours and body weight were measured three times weekly. Measurements were entered directly into an

© 1999 Cancer Research Campaign

**Table 2** Slopes of the linear regression analyses of mouse body weights (g change per day) following dietary modification and CPT-11 treatment[a]

| Diet | Days 1–14 after dietary modification (mean ± SD) | Days 14–33 after dietary modification (mean ± SD) |
|---|---|---|
| Control, no CPT-11 | –0.049 ± 0.028 | no CPT-11, 0.22 ± 0.024 |
| 7% Corn oil | –0.010 ± 0.042 | with CPT-11, –0.047 ± 0.020 |
| 3% Fish oil | 0.064 ± 0.048 | with CPT-11, –0.116 ± 0.020 |
| 6% Fish oil | 0.038 ± 0.036 | with CPT-11, –0.067 ± 0.016 |

[a]During days 1–14, no slope was significantly different from a slope of zero (no change in body weight per day). During the time of CPT-11 treatment (60 mg $kg^{-1}$ body weight CPT-11 each 4 days) all CPT-11-treated groups showed significant weight loss. ANOVA followed by SNK multiple range test of the slopes showed that there was no significant difference in the slope (weight loss) due to the diet of the CPT-11-treated mice.

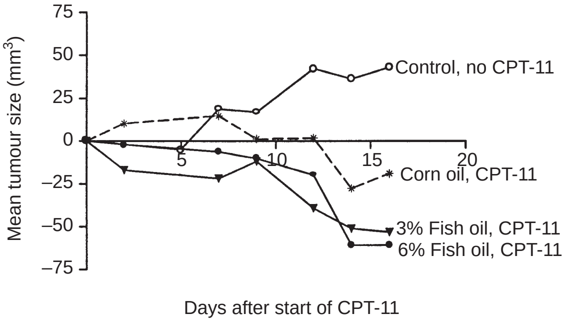

**Figure 1** Growth of MCF7 human breast cancer xenografts in nude mice. Mice with growing tumours were divided into four groups (5 mice per group) and fed chow (control, no CPT-11) or modified AIN-76A diets containing 7% corn oil; 3% fish oil with 4% corn oil; or 6% fish oil with 1% corn oil. After 2 weeks on the diet, CPT-11 treatment (60 mg/kg body weight, i.v. q4d × 6) was initiated. The mean tumour volume for each group was normalized to zero at the beginning of the CPT-11 treatment. Mice were killed 5 days after the sixth injection of CPT-11. The results of statistical analyses of the change in tumour size are reported in the text

Excel spreadsheet. Tumour sizes were calculated using the formula for the volume of a prolate spheroid: V = 4/3 * 3.14 * L/2 *W/2 * D/2. The width measurement was used as the depth of the tumour. This shape was a good approximation of the shape of the tumours.

The experiment was terminated 28 days after the initiation of CPT-11 treatment. The mice were anaesthetized using a ketamine/S. A. rompun mixture (0.2 cc per 25 g weight, IM; prepared by our Laboratory Animal Resources veterinarian), then killed by cervical dislocation.

### Necropsy and tissue processing

Tumours, liver, small and large intestines were removed at necropsy. Tumours were fixed in Omnifix® (Melville, NY, USA) for later analysis. Omnifix® is an alcohol-based, proprietary formula fixative which does not cross-link antigen epitopes as does formalin. A portion of the liver was flash frozen in liquid nitrogen for later analyses. A 1-cm segment of each small intestine and a 1-cm segment of each large intestine were consistently removed from regions 2 cm from the stomach/duodenum junction or 4 cm from the anus respectively. These tissue segments were placed on a small piece of cardstock, split longitudinally, spread and affixed mucosal side up to the card. Card and tissue were then placed in Omnifix® for fixation. After fixation, tissues from individual mice were placed in a tissue cassette and processed for embedding in paraffin blocks. Small and large intestine segments were oriented on-edge in the paraffin blocks so that complete longitudinally sectioned crypts would be seen on microscope slides. Four-micrometer-thick sections were cut and placed on slides. One set of slides was stained using haematoxylin and eosin (H&E), a second set of slides was stained by the periodic acid-Schiff (PAS) reaction and counterstained with haematoxylin to identify mucin in goblet cells. Slides were coded and evaluated by an observer blinded to the group of origin of each slide.

### Products of lipid peroxidation

At a later date, frozen livers were thawed and homogenized individually at 4°C using a Polytron homogenizer. The total protein content of an aliquot of the whole specimen homogenate was analysed by the method of Bradford (Bradford, 1976) using the Bio-Rad protein assay (micro-method). The thiobarbituric acid reactive substances (TBARS) assay was used to estimate lipid peroxidation on the remainder of the homogenate. Malondialdehyde and other products of lipid peroxidation can be estimated spectrophotometrically at 535 nm after reaction with thiobarbituric acid to obtain an index for lipid peroxidation (Esterbauer et al, 1991). We realize that TBARS does not measure all products of lipid peroxidation and that there may be minor interference by other substances (sugars, amino acids, etc.), however, this simple inexpensive test does provide a good estimate of changes in overall lipid peroxidation of tissues. The absorbance values obtained were compared against a standard curve of known concentrations of malondialdehyde and normalized by protein content of the specimen. The results were reported as nmol of TBARS per mg of protein.

### Histological analyses of duodenum and colon

Only complete midaxially sectioned crypts in duodenum and colon on H&E-stained slides were selected for analyses of crypt height, and number and location of mitotic and apoptotic cells. Complete crypts were defined as those with:

1. the crypt base at the muscularis mucosa
2. an open lumen from mouth to base
3. a single column of epithelial cells up each side of the crypt.

Crypt height was defined as the number of cells in a single column from the centre of the base to the mouth of the crypt in complete midaxially sectioned crypts. Mitotic and apoptotic figures in the duodenum were identified on H&E-stained slides. The position of each mitotic or apoptotic figure in number of cells from the centre of the crypt base were recorded. Apoptotic events were identified by the morphological parameters of nuclear marginalization of the chromatin, condensation of the cytoplasm, cell shrinking, membrane blebbing and, finally, fragmentation of the cell into apoptotic bodies (Potten, 1992; Barnes et al, 1997).

PAS-stained slides were used to determine the distribution of mucin containing goblet cells. Complete, midaxially sectioned crypts in sections of duodenum and colon were identified then the location of each PAS-stained cell ($PAS^+$) in number of cells from the centre of the crypt base to the crypt mouth was recorded.

 © 1999 Cancer Research Campaign

The thickness of the muscularis mucosa was measured on the H&E-stained sections using a calibrated ocular micrometer.

### Statistical analyses

Mean body weights during the experiment were analysed by linear regression analyses using PRISM®. Body weight data were divided into two linear regression analyses: (1) the time before the initiation of CPT-11 and (2) the time after initiation of CPT-11 to determine the effect of the diet and of the CPT-11 on the rate of change of the mean body weights of each group of mice. Significant differences between the slopes of the linear regression analyses either before or after CPT-11 treatment were determined by analysis of variance (ANOVA) followed by a Student–Newman–Keuls (SNK) multiple range test using PRISM® software.

The mean tumour volume for each group was normalized to zero at the beginning of the CPT-11 treatment. Mean tumour growth curves were generated for each group and linear regression analysis was used to assess the tumour growth rate of each group. A significant positive slope indicated tumour growth, a significant negative slope indicated tumour regression and a non-significant slope indicated no growth. Analyses for differences between slopes of the regressions of the mean tumour volumes of each group were performed by PRISM® (Graphpad Software, San Diego, CA, USA) using the general linear model to generate an ANOVA. An SNK multiple range test was used to determine which slopes were significantly different against the null hypothesis that there was no difference between the slopes. A probability value ($P$) ≤ 0.05 was used to indicate that the tumour growth rates represented by the slopes were significantly different.

ANOVA followed by SNK was used to determine differences between groups in TBARS in the livers.

Kolmogorov–Smirnov (K–S) tests for normality showed that the distributions of the heights of mitotic and apoptotic figures in duodenum crypts differed significantly from a normal distribution. However, the distribution of the square root transformed heights of mitotic and apoptotic figures did not differ significantly from a normal distribution. Thus, a parametric ANOVA followed by an SNK was used to test for differences between the means of the square root transformed heights of mitotic figures or of apoptotic figures.

The K-S test showed that the crypt heights and the locations of PAS-positive cells along the length of the crypt were not significantly different from a normal distribution, thus transformation was not needed prior to ANOVA of crypt heights or of means of locations of PAS-positive cells. A $P \le 0.05$ was used to indicate that the means of two groups were significantly different.

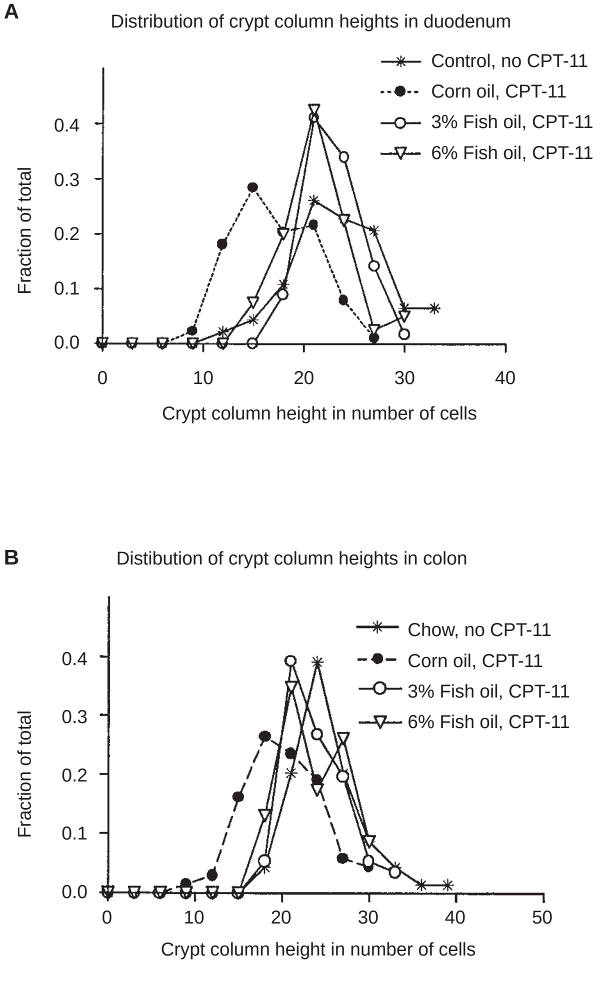

**Figure 2** Influence of supplementation of the diet with fish oil on alteration in the distribution of intestinal crypt column heights in the duodenum (**A**) and in the descending colon (**B**) by CPT-11 treatment. Results of the statistical analyses of these distributions are reported in Table 3

### RESULTS

### Mouse body weights

The slopes of the linear regression analyses (Table 2) of the mean body weights (rate of change in body weight) per group for days 1–14 (prior to any drug treatment) were determined to provide information about the effect of diet on the mouse body weight. The effect of treatment with CPT-11 on body weight was determined from the mean body weights for days 14–42 of the study. Results are shown in Table 2.

### Mean tumour size

The mean tumour sizes over the time of treatment are presented in Figure 1. The slope of the linear regression analyses (tumour growth rate in $mm^3$ $day^{-1}$) for the control group which did not receive CPT-11 was significantly positive (indicating continued tumour growth, slope = 3.2 ± 0.7) and was significantly different from the tumour growth rates of the three groups of mice which received CPT-11. The tumour growth rate of the group of mice treated with CPT-11 and fed 7% corn oil (slope = –1.8 ± 0.8) was not significantly different from a slope of zero (indicating that growth of the tumour was halted by the CPT-11 treatment in this group of mice) and the tumour growth rate in the 7% corn oil fed group was significantly different from that of the other three groups. The mean tumour growth rates of the groups treated with CPT-11 and fed 3% fish oil (slope = –3.1 ± 0.6) or 6% fish oil (slope = –3.9 ± 1.0) were:

1. significantly negative, indicating significant regression of the tumour
2. not significantly different from each other
3. significantly different from the mean tumour growth rate of the mice which did not receive CPT-11

© 1999 Cancer Research Campaign

**Table 3** Influence of dietary fish oil on alteration of intestinal crypt column height[a] by CPT-11 treatment

| Diet 5 mice per group | CPT-11 | $n^b$ | Duodenum mean ± s.d. | $n^b$ | Colon mean ± s.d. |
|---|---|---|---|---|---|
| Chow | – | 92 | 23.5 ± 5.0[d] | 70 | 25.0 ± 4.0[c] |
| 7% Corn oil | + | 90 | 17.1 ± 4.0 | 66 | 20.1 ± 4.5 |
| 3% Fish oil | + | 74 | 22.6 ± 2.6[d] | 56 | 23.7 ± 3.3[c] |
| 6% Fish oil | + | 64 | 21.0 ± 3.2[c] | 23 | 23.3 ± 3.8[c] |

[a]Crypt column height is expressed in number of cells from the base to the mouth of the crypt. [b]*n* = total number of crypt columns counted per group. [c,d]ANOVA followed by SNK multiple range test showed that means which share a superscript in a column are not significantly different.

4. significantly different from the mean tumour growth rate of the mice which were treated with CPT-11 and fed 7% corn oil.

### Thiobarbituric acid reactive substances

ANOVA revealed that when mice were killed 5 days after treatment with CPT-11, there was not a significant difference in the nmol TBARS $mg^{-1}$ protein in the livers of the mice due to treatment with CPT-11 or to the diet of the mice (data not shown). Tumours of the CPT-11 treated and fish oil fed groups had regressed to the extent that there was not enough tumour tissue for TBARS analyses.

### Histomorphometric analyses of duodenum and colon

### *Crypt column heights*

Graphs of the distributions of the crypt heights in the duodenum (Figure 2A) and colon (Figure 2B) illustrate that the mean of the distribution of crypt heights of the group treated with CPT-11 and fed 7% corn oil was less than the means of the other three groups. The distributions of crypt heights of the groups treated with CPT-11 and fed either 3% or 6% fish oil were similar to that of the group which did not receive CPT-11 treatment.

ANOVA followed by SNK of the crypt column height data (Table 3) revealed that, when compared to the control group which did not receive CPT-11:

1. the mean crypt column heights in the duodenum and colon were significantly less in mice fed 7% corn oil and treated with CPT-11
2. the mean crypt column heights in the duodenum and colon were not significantly less in mice fed 3% fish oil and treated with CPT-11
3. the mean crypt column height in the colon was not significantly less in mice fed 6% fish oil and treated with CPT-11.

ANOVA also revealed that the crypt column heights in both the duodenum and colon of mice fed either 3% or 6% fish oil and treated with CPT-11 were significantly greater than in mice fed 7% corn oil and treated with CPT-11.

### Mitotic figures

ANOVA of the numbers of mitotic figures per midaxial crypt section in the duodenum (Table 4) revealed that there was not a significant difference between groups. However, ANOVA followed by SNK of the square root transformed heights of the mitotic figures revealed that there was a significant difference between groups in the distribution of mitotic figures in the duodenum. Specifically, the mean of the distribution of the heights of mitotic figures in the duodenum of the group treated with CPT-11 and fed corn oil was significantly less than the mean of the distribution in the mice which did not receive CPT-11 or in the mice treated with CPT-11 and fed 3% fish oil. The distribution of mitotic figures in the duodenum of the groups which were treated with CPT-11 and fed either 3% or 6% fish oil was similar to the group which did not receive CPT-11. There were too few mitotic figures in the histologic sections of colon for meaningful statistical analysis.

### Apoptotic figures

ANOVA followed by SNK revealed that the number of apoptotic figures per midaxial crypt section in the duodenum of the group treated with CPT-11 and fed corn oil was significantly higher than in the control group which did not receive CPT-11 (Table 4). The mean number of apoptotic figures in the groups treated with CPT-11 and fed either 3% or 6% fish oil was intermediate and not significantly different from either the control group which did not receive CPT-11 or the group treated with CPT-11 and fed corn oil.

The mean height of the distribution of apoptotic figures in the duodenum of the group treated with CPT-11 and fed corn oil was significantly less than the mean of that distribution in the mice which did not receive CPT-11 or in the mice treated with CPT-11 and fed 3% fish oil, as summarized in Table 4. There were too few apoptotic figures in the histologic sections of colon for meaningful statistical analysis.

**Table 4** Influence of CPT-11 treatment dietary fish oil on the number and the location of mitotic and apoptotic figures in duodenum crypts

| Diet 5 mice/per group | CPT –11 | N mitotic figures[a] | Mitotic figures per crypt[b] | Trans. height mitotic figures[b] | N apoptotic figures[a] | Apoptotic figures per crypt[b] | Trans. height apoptotic figures[c] |
|---|---|---|---|---|---|---|---|
| Chow | – | 101 | 2.2 ± 1.5[d] | 3.1 ± 0.9[d] | 52 | 0.46 ± 0.19[d] | 2.79 ± 0.95[d] |
| Corn oil, | + | 93 | 2.1 ± 1.5[d] | 2.5 ± 0.7[e] | 58 | 1.14 ± 0.49[e] | 2.03 ± 0.76[e] |
| 3% Fish oil | + | 73 | 2.6 ± 1.7[d] | 2.9 ± 0.8[d] | 88 | 0.74 ± 0.22[de] | 2.76 ± 0.81[d] |
| 6% Fish oil | + | 40 | 2.0 ± 0.9[d] | 2.6 ± 0.6[e] | 80 | 0.90 ± 0.28[de] | 2.30 ± 0.83[e] |

[a]Total number of mitotic or apoptotic figures counted per group. [b]Mean number of mitotic or apopotic figures per midaxial crypt section (mean ± s.d.). [c]Mean of the square root transformed heights of mitotic or apoptotic figures (mean ± s.d.). [de]Means that share the same superscript in a column are not significantly different.

 © 1999 Cancer Research Campaign

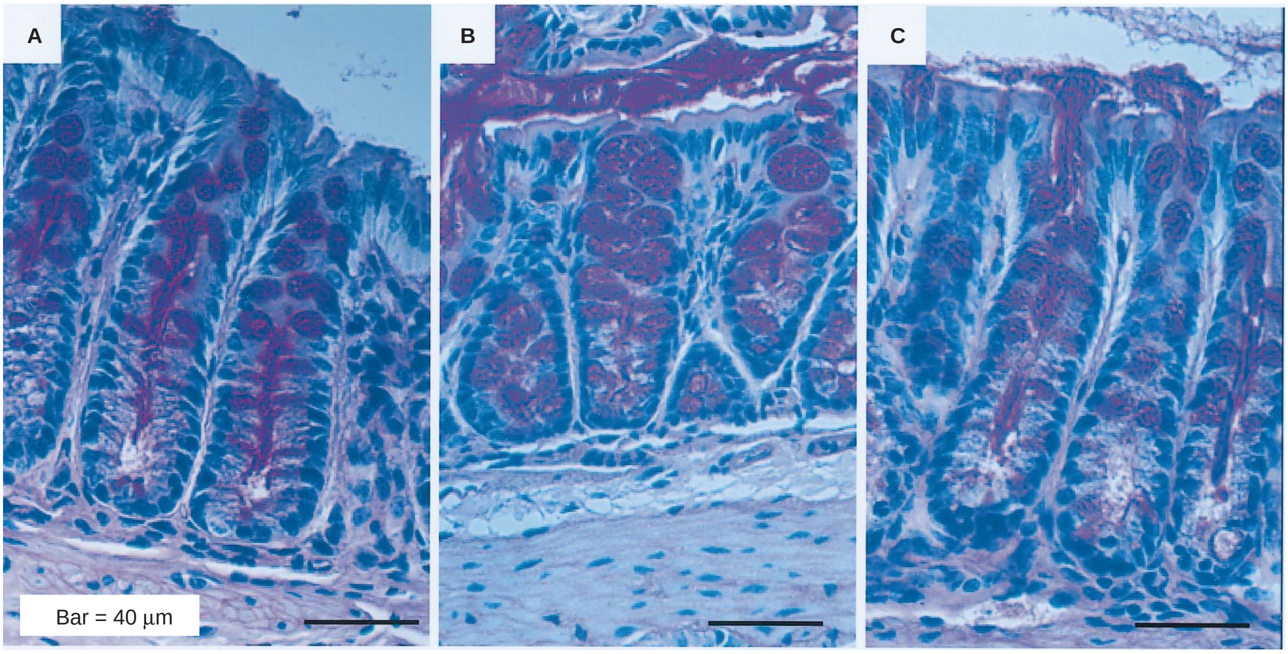

**Figure 3** Photomicrographs of histologic sections of the descending colon mucosa of female nude (*nu/nu*) mice. Sections were stained by the periodic acid-Schiff (PAS) reaction and counterstained with haematoxylin. The crypts pictured are representative of crypts from mice fed: (**A**) the mouse chow diet and not treated with CPT-11; (**B**) the 7% corn oil diet for 2 weeks prior to and during the course of CPT-11 treatment (60 mg $kg^{-1}$ body weight, i.v. q4d × 6); (**C**) the 3% fish oil and 4% corn oil diet prior to and during the same course of CPT-11 treatment. Mice were killed 5 days after the last injection of CPT-11. The magnification of each photomicrograph is the same allowing visual comparison of crypt heights and of the distribution and size of the $PAS^+$ goblet cells. Notice that crypts are shorter and that there is goblet cell hyperplasia in the photomicrograph of a colon section from a mouse fed 7% corn oil diet and treated with CPT-11 (4-µm-thick sections, bar = 40 µm)

**Table 5** Influence of dietary fish oil on alteration of number and distribution of $PAS^+$ goblet cells in descending colon crypts by CPT-11 treatment

| | | **Descending colon** | | | **Duodenum** | | |
|---|---|---|---|---|---|---|---|
| **Diet *n*=5 mice/group** | **CPT –11** | **Number goblet cells scored** | **Mean (± s.d.) goblet cells/ per crypt column[a]** | **Mean (± s.d.) height of goblet cells in crypt** | **Number goblet cells scored** | **Mean (± s.d.) goblet cells per crypt column[a]** | **Mean (± s.d.) height of goblet cells in crypt** |
| Chow | – | 88 | 5.5 ± 1.8[c] | 16.4 ± 6.2[b] | 73 | 2.4 ± 1.3[b] | 13.6 ± 6.0[b] |
| 7% Corn oil | + | 107 | 8.2 ± 2.7[b] | 13.1 ± 6.2[c] | 30 | 1.3 ± 0.6[c,d] | 12.1 ± 3.7[b,c] |
| 3% Fish oil | + | 89 | 5.6 ± 1.9[c] | 13.2 ± 6.3[c] | 40 | 1.7 ± 1.3[c] | 11.4 ± 4.2[b,c] |
| 6% Fish oil | + | 78 | 5.3 ± 2.4[c] | 11.5 ± 5.5[c] | 24 | 1.0 ± 1.0[d] | 10.3 ± 3.6[c] |

[a]Mean number of goblet cells per crypt column scored in midaxially sectioned crypts. [b,c,d]Column means that share the same superscript are not significantly different.

### $PAS^+$ goblet cells

ANOVA followed by SNK revealed that the mean number of $PAS^+$ goblet cells per colon crypt column in the group of mice treated with CPT-11 and fed the 7% corn oil diet (Table 5) was significantly higher than in the group of mice which did not receive CPT-11. However, in the groups of mice fed the 3% or 6% fish oil diet and treated with CPT-11, the mean number of $PAS^+$ goblet cells per colon crypt was not significantly different from that of the control group of mice which did not receive CPT-11. Examples of these findings are depicted in the photomicrographs in Figure 3. The mean height of $PAS^+$ goblet cells in the colon crypts was significantly lower in all groups of mice treated with CPT-11 than in the group of mice not treated with CPT-11.

Statistical analyses by ANOVA followed by SNK revealed that in groups of mice treated with CPT-11, the mean number of goblet cells per crypt column in the duodenum (Table 5) was significantly less than in the mice which did not receive CPT-11. However, the mean height of $PAS^+$ goblet cells in the duodenum crypts of mice fed 7% corn oil or 3% fish oil diets and treated with CPT-11 was not significantly different from that of the control group of mice which did not receive CPT-11.

### Thickness of muscularis mucosa

Statistical analysis by ANOVA followed by SNK revealed that the thickness of the muscularis mucosa layer in the colons of mice treated with CPT-11 and fed the diet containing 6% fish oil

© 1999 Cancer Research Campaign

(10.8 ± 0.2 μm) was not significantly different from that of the mice not treated with CPT-11 (11.1 ± 0.3 μm). However, the thickness of the muscularis mucosa layer in the colons of mice fed 7% corn oil and treated with CPT-11 (6.6 ± 0.2 μm) was significantly less than in the mice not treated with CPT-11 or the mice fed 6% fish oil and treated with CPT-11. The thickness of the muscularis mucosa layer in the colons of mice fed 3% fish oil and treated with CPT-11 (7.7 ± 0.2 μm) was intermediate in thickness but was not significantly different from the mean value of the other groups. The muscularis mucosa layer in the duodenum of all groups was too thin for reliable measurements.

### DISCUSSION

Results from the present study demonstrate that 60 mg CPT-11 per kg body weight (i.v. q4d × 6) halted the growth of, but did not cause significant regression of, MCF7 human breast carcinoma xenografts in groups of mice fed a diet containing 7% corn oil. However, when the diet of the mice was supplemented with 3% or 6% fish oil, the same CPT-11 treatment caused a significant regression of the MCF7 xenograft. All of the tumours in the mice fed fish oil regressed to the extent that there was not sufficient tumour tissue to investigate mechanisms contributing to the regression (i.e. lipid peroxidation) as we did in earlier studies (Hardman et al, 1997). Because of this fact, we could not test the hypothesis that the combination of dietary fish oil and CPT-11 administration leads to the selective accumulation of lipid peroxidation products to cytotoxic levels in the MCF7 xenograft. As in the earlier study (Hardman et al, 1997), dietary fish oil did not significantly increase potentially cytotoxic lipid peroxidation products in the host liver.

In addition to enhancing the efficacy of CPT-11 against growth of MCF7 human breast carcinoma xenografts, supplementation of the diet with 3% or with 6% w/w fish oil resulted in amelioration of the intestinal damage resulting from CPT-11 treatment. To evaluate the intestinal damage resulting from CPT-11 treatment, we examined intestinal mucosa taken from mice treated with CPT-11 (60 mg $kg^{-1}$ q4d × 6) and killed 5 days after the last injection. The dosage of CPT-11 used in our study was less than that used in prior studies and the tissues were removed for study at a longer duration after the last dose of CPT-11 than in prior studies (Ikuno et al, 1995; Cao et al, 1998; Shinohara et al, 1998). Thus, we examined longer term changes in intestinal mucosa following administration of more clinically relevant doses than previous studies. In mice treated with CPT-11 and fed the 7% corn oil diet, we found that there was a significant ($P < 0.05$) shortening in crypt heights in the duodenum accompanied by a significant decrease in number of goblet cells, a significant increase in number of apoptotic cells and a significant decrease in the size (height) of the cell proliferation compartment compared to mice which did not receive CPT-11. Increased apoptotic death in the CPT-11 treated mice fed corn oil, which was not accompanied by increased mitotic activity in the crypt epithelium, may help account for the decreased number of cells in the duodenum crypt column.

In the colon of CPT-11 treated mice fed 7% corn oil, the muscularis mucosa was significantly thinner, the mean crypt column height was significantly shorter and the shorter column height was accompanied by a significant increase in number of goblet cells than in the colon of mice not treated with CPT-11. Specifically, the number of goblet cells in the colon crypt column increased by 49% (hyperplasia) but the number of non-goblet cells (mainly columnar absorptive cells) was decreased by 39% following treatment with CPT-11. The reduction in the number of columnar absorptive cells in the colon may help account for the reported malabsorption of water and electrolytes in the large bowel of CPT-11-treated individuals (Kase et al, 1997*a*).

Not only were there more goblet cells in the colon crypt column of mice treated with CPT-11 and fed corn oil than in those which did not receive CPT-11, but the distribution of goblet cells was shifted towards the base of the colon crypts and the goblet cells tended to be larger as illustrated in Figure 3. The presence of larger goblet cells indicates that CPT-11 treatment caused accumulation of mucous secretory granules in the goblet cells. Increased accumulation of mucus could be due to a failure of the goblet cells to secrete the mucous granules and/or due to an increased synthesis of mucus. Regardless, we can state that a diet which included fish oil prevented CPT-11-induced goblet cell hyperplasia in the colon but did not prevent the shift of the distribution of goblet cells towards the base of colon crypts.

In a past report (Ikuno et al, 1995), goblet cell hyperplasia in the cecum was attributed to induction of differentiation of goblet cells by CPT-11. Our findings of goblet cell *hypo*plasia in the duodenum of mice fed 7% corn oil indicates that CPT-11 failed to induce a greater number of differentiated goblet cells in the duodenum, thus the cells of the small and large intestine responded differently to CPT-11 treatment. Further research is needed to understand the effect of CPT-11 treatment on goblet cell differentiation and the effect of the alteration in the goblet cell population on colon function.

Taken together, the histological changes in muscularis mucosa thickness, numbers of absorptive and goblet cells per midaxial crypt column and goblet cell size, as described here, indicate that CPT-11 treatment induced structural changes in the intestinal mucosa of mice fed 7% corn oil which persisted at least 5 days following the last dose of CPT-11. On the other hand, the intestinal mucosal architecture of mice that were treated with CPT-11 and fed the diets containing fish oil was largely the same as the intestinal mucosal architecture of the mice not treated with CPT-11.

Diarrhoea is a dose-limiting side-effect of CPT-11 treatment, thus, attempts have been made to understand the causes of the severe diarrhoea induced by CPT-11 treatment (Sawada et al, 1991; Sakai et al, 1995, 1997; Rothenberg, 1996; Kase et al, 1997*b*; Shinohara et al, 1998). There are various causes of diarrhoea including: hyperperistalsis, malabsorption of water and electrolytes and/or hypersecretion of $Cl^-$. Although the exact mechanism of CPT-11-induced diarrhoea is not defined, several approaches have been explored to overcome the toxicity of CPT-11 to the intestines, including:

1. The use of the bacterial lipopeptide (JBT 3002), to stimulate intestinal mucosal immunity and to help maintain mucosal architecture (Shinohara et al, 1998). In this regard, Shinohara et al (1998) reported that treatment of mice with CPT-11 (i.p. 100 mg $kg^{-1}$ q4d) was effective against liver metastasis of the CT-26 murine colon cancer in a syngeneic mouse model. They also reported that oral administration of JBT 3002 prior to the same treatment with CPT-11 resulted in: increased inhibition of liver metastasis, reduced mouse mortality and reduced damage to the mouse intestines (Shinohara et al, 1998) and proposed that the mechanism of action of JBT 3002 was via its modulation of immune activity. Likewise, Cao et al (1998) demonstrated that use of interleukin 15 offered protection against CPT-11 induced intestinal damage and suggested that

 © 1999 Cancer Research Campaign the mechanism for protection was by immunomodulation (Cao et al, 1998).
2. The inhibition of thromboxane $A_2$ ($TXA_2$) synthesis, blocking of $TXA_2$ receptors (Sakai et al, 1997) or the use of various drugs to inhibit prostaglandins $E_2$ ($PGE_2$) and $F_2$ ($PGE_2$) to inhibit the ability of these eicosanoids to stimulate $Cl^-$ secretion and decrease water absorption in the colon (Sakai et al, 1995; Kase et al, 1997*b*).
3. The use of an inhibitor of the conversion of CPT-11 to SN-38, its more active metabolite (Narita et al, 1993).
4. The use of drugs including atropine or loperamide. Atropine decreases intestinal motility (peristalsis) by blocking the activity of acetylcholine (Takasuna et al, 1995; *Mosby's Complete Drug Reference*, 1997*a*). Loperamide acts to slow intestinal motility and affects water and electrolyte movement through the bowel (*Mosby's Complete Drug Reference*, 1997*b*). High-dose loperamide has been used effectively to treat CPT-11-induced diarrhoea (Abigerges et al, 1994). However, loperamide at 50 μmol $l^{-1}$ has been shown to reduce the conversion of CPT-11 to SN-38 (the active metabolite) in vitro which may interfere with CPT-11 anticancer action (Rivory et al, 1996*b*). The effect of loperamide on the efficacy of CPT-11 in vivo is not known (Rivory, 1996*a*).
5. The use of antibiotics to reduce the effects of infection and loss of intestinal integrity and normal function (Takasuna et al, 1996).

To this list, we now add use of dietary fish oil supplementation. The amount of fish oil needed to normalize intestinal mucosal structure and function and how fish oil may work are discussed in the next two sections.

### Relevance of consumption by mice of 3% or 6% w/w dietary fish oil to humans

It has been reported (Anti et al, 1992; Horrobin, 1994) that adult humans can consume about 10–12 g of fish oil per day without adverse side-effects. We have personally verified that taking this amount of encapsulated fish oil per day for at least 2 months caused no ill effects. How does consumption of 10–12 g fish oil per day by humans compare to the 3% and 6% fish oil diets fed to the mice in this study?

At nine calories per g of fat, 10 g of fish oil contains 90 calories. Therefore for a person consuming 1800 calories per day, 10 g of fish oil in the diet would be 90/1800 = 5% of calories from fish oil. Each 100 g of the 7% fat mouse diet supplied 395 calories. Therefore, in the 3% w/w fish oil diet, 3 g per 100 g of the diet was fish oil. It follows that fish oil accounted for 27/395 or 6.8% of calories in the mouse diet. These calculations show that the 3% fish oil diet is in a range which humans can easily consume by supplementation of the diet with 10–12 g of fish oil per day.

### Relevance of fish oil supplementation on prostaglandin synthesis in colon

Increased concentrations of $PGE_2$ and $TXA_2$ in the large bowel have been associated with increased $Cl^-$ secretion and diarrhoea resulting from CPT-11 administration (Kase et al, 1997*b*; Sakai et al, 1997). The types of eicosanoids produced by cells are readily modified based on the precursor fatty acids which are available for eicosanoid synthesis (Weber and Sellmayer, 1990). When n-3 fatty acids are consumed in the diet, eicosapentanoic acid, which is the substrate for synthesis of n-3 derived eicosanoids such as $PGE_3$ and $TXA_3$, is proportionately increased in the phospholipids of cellular membranes (Anti et al, 1992) while arachidonic acid, which is the substrate for synthesis of n-6 derived eicosanoids such as $PGE_2$ and $TXA_2$, is decreased. Indeed, as little as 4.4 g of n-3 fatty acids per day significantly increased EPA, decreased arachidonic acid and decreased the $PGE_2$ in colonic mucosa of healthy volunteers (Bartram et al, 1993). Thus suppression of $PGE_2$ and $TXA_2$ synthesis by consumption of dietary n-3 FA would be expected to decrease the diarrhoea induced by eicosanoid mediated Cl–secretion and would be expected to decrease the diarrhoea associated with CPT-11 treatment. Evaluation of the effect of dietary n-3 fatty acid consumption on stimulation of eicosanoid synthesis as a result of CPT-11 treatment warrants further investigation.

### Implication

Regardless of the need for additional research on the specific mechanism for activity, we believe that the results of our current preclinical trial, using dietary fish oil supplementation in combination with CPT-11, are sufficient to recommend the initiation of clinical testing of dietary supplementation with fish oil as an effective, non-toxic and inexpensive method to decrease or prevent severe diarrhoea in cancer patients and to increase the efficacy of the anticancer drug, CPT-11.

### ACKNOWLEDGEMENTS

This work was supported by the American Institute for Cancer Research, the Cancer Research Foundation of America, a Small Business Technology Transfer grant from the National Institutes of Health (R41CA76763-01) and a NCI Cancer Center Support grant (P30CA54174). CPT-11 was a gift from Pharmacia and Upjohn, Kalamazoo, MI, USA.

#### REFERENCES

Abigerges D, Armand J-P, Chabot GG and et al (1994) Irinotecan (CPT-11) high-dose escalation using intensive high-dose loperamide to control diarrhea. *J Natl Cancer Inst* **86**: 446–449

Anti M, Marra G, Armelao F, Bartoli GM, Ficarelli R, Percesepe A, De Vitis I, Maria G, Sofo L, Rapaccini GL, Gentiloni N, Piccioni E and Miggiano G (1992) Effect of Ω-3 fatty acids on rectal mucosal cell proliferation in subjects at risk for colon cancer. *Gastro* **103**: 883–891

Barnes CJ, Lee M, Hardman WE and Cameron IL (1997) NSAID modulation of colonic epithelial cell proliferation and apoptosis as intermediate biomarkers of induced rat colon cancer. *Br J Cancer* **77**: 573–580

Bartram H, Gostner A, Scheppach W, Reddy BS, Rao CV, Dusel G, Richter F, Richter A and Kasper H (1993) Effects of fish oil on rectal cell proliferation, mucosal fatty acids, and prostaglandin E2, release in healthy subjects. *Gastro* **105**: 1317–1322

Bradford MM (1976) A rapid and sensitive method for the quantitation of microgram quantities of protein utilizing the principle of protein-dye binding. *Anal Biochem* **72**: 248–254

Cao S, Black JD, Troutt AB and Rustum YM (1998) Interleukin 15 offers selective protection from irinotecan-induced intestinal toxicity in a preclinical animal model. *Cancer Res* **58**: 3270–3274

Colvin OM, Cokgor I, Ashley DM, Kerby T, Arbuck S, Malczyn J, Miller L, Cloughesy T, Houghton PJ, Rich J, Friedman AH and Friedman HS (1998) Irinotecan treatment of adults with recurrent or progressive malignant glioma. *Proc Am Soc Clin Oncology* **16**: (Abstract)

Esterbauer H, Schaur RJ and Zollner H (1991) Chemistry and biochemistry of 4-hydroxynonenal, malondialdehyde and related aldehydes. *Free Rad Biol Med* **11**: 81–128

*© 1999 Cancer Research Campaign* 

Gerrits CJH, de Jonge MJA, Schellens JHM, Stoter G and Verweij J (1997) Topoisomerase I inhibitors: the relevance of prolonged exposure for present clinical development. *Br J Cancer* **76**: 952–962

Hardman WE, Barnes CJ, Knight CW and Cameron IL (1997) Effects of iron supplementation and ET-18-$OCH_3$ on MDA-MB 231 breast carcinomas in nude mice consuming a fish oil diet. *Br J Cancer* **76**: 347–354

Horrobin DF (1994) Unsaturated lipids and cancer. In: *New Approaches to Cancer Treatment: Unsaturated Lipids and Photodynamic Therapy*, Horrobin DF (ed), pp. 3–29. Churchill Livingstone: London

Ikuno N, Soda H, Watanabe M and Oka M (1995) Irinotecan (CPT-11) and characteristic mucosal changes in the mouse ileum and cecum. *J Natl Cancer Inst* **87**: 1876–1883

Kase Y, Hayakawa T, Aburada M, Komatsu Y and Kamataki T (1997*a*) Preventive effects of *Hange-shashin-to* on irinotecan hydrochloride-caused diarrhea and its relevance to the colonic prostaglandin $E_2$ and water absorption in the rat. *Jpn J Pharmacol* **75**: 407–413

Kase Y, Hayakawa T, Togashi Y and Kamataki T (1997*b*) Relevance of irinotecan hydrochloride diarrhea to the level of prostaglandin $E_2$ and water absorption of large intestine in rats. *Jpn J Pharmacology* **75**: 399–405

Kawato Y, Aonuma M, Hirota Y, Kuga H and Sato K (1991) Intracellular roles of SN-38, a metabolite of the camptothecin derivative CPT-11, in the antitumor effect of CPT-11. *Cancer Res* **51**: 4187–4191

*Mosby's Complete Drug Reference 1997 Physicians GenRx-Drug Information*, Atropine sulfate. II-175-II-177. Edited by Publisher: Ladig D. Mosby-Year Book: St Louis

*Mosby's Complete Drug Reference 1997 Physicians GenRx-Drug Information*, Loperamide hydrochloride. II-1286-II-1287. Edited by Publisher: Ladig D. Mosby-Year Book: St Louis

Narita M, Nagai E, Hagiwara H, Aburada M, Yokoi T and Kamataki T (1993) Inhibition of β-glucoronidase by natural glucuronides of kampo medicines using glucuronide of SN-38 (7-ethyl-10-hydroxycamptothecin) as a substrate. *Xenobiotica* **23**: 5–10

Potten CS (1992) The significance of spontaneous and induced apoptosis in the gastrointestinal tract of mice. *Cancer Metast Rev* **11**: 179–195

Rivory LP (1996) Irinotecan (CPT-11): a brief overview. *Clin and Exp Pharmacol and Physiol* **23**: 1000–1004

Rivory LP, Bowles MR, Robert J and Pond SM (1996) The conversion of CPT-11 (irinotecan) to its active metabolite, SN-38, by human liver carboxylesterase. *Biochem Pharmacol* **52**: 1103–1111

Rothenberg M (1996) The current status of irinotecan (CPT-11) in the United States. *Ann NY Acad Sci* **803**: 272–281

Sadzuka Y and Hirota S (1997) Effect of CPT-11 on lipid peroxidation level in mouse tissues. *Jpn J Cancer Res* **88**: 512–516

Sakai H, Diener M, Gartmann V and Takeguchi N (1995) Eicosanoid-mediated $Cl^-$secretion induced by the antitumor drug, irinotecan (CPT-11), in the rat colon. *Naunyn-Schmiedeberg's Arch Pharmacol* **351**: 309–314

Sakai H, Sato T, Hamada N, Yasue M, Ikari A, Kakinoki B and Takeguchi N (1997) Thromboxane $A_2$, released by the anti-tumour drug irinotecan, is a novel stimulator of $Cl^-$secretion in isolated rat colon. *J Physiol* **505.1**: 133–144

Sawada S, Okajima S, Aiyama R, Nokota K, Furuta T, Yokokura T, Sugini E, Yamaguchi K and Miyasaka K (1991) Synthesis and antitumor activity of 20(S)-camptothecin derivatives. Carbamate-linked, water-soluble derivatives of 7-ethyl-10-hydrocamptothecin. *Chem Pharm Bull* **39**: 1446–1454

Shinohara H, Killion J, Kuniyasu H, Kumar R and Fidler IJ (1998) Prevention of intestinal toxic effects and intensification of irinotecan's therapeutic efficacy against murine colon cancer liver metastases by oral administration of the lipopeptide JBT 3002. *Clin Cancer Res* **4**: 2053–2063

Takasuna K, Kasai Y, Kitano Y, Kakihata K, Hirohashi M and Nomura M. (1995) Study on the mechanisms of diarrhea induced by a new anticancer camptothecin derivative, irinotecan hydrochloride (CPT-11) in rats. *Folia Pharmacol Jpn* **105**: 447–460

Takasuna K, Hagiwara T, Kato M, Namoru M, Nagai E, Yokoi T and Kamataki T (1996) Involvement of β-glucuronidase in intestinal microflora in the intestinal toxicity of the antitumor camptothecin derivative irinotecan hydrochloride (CPT-11) in rats. *Cancer Res* **56**: 3752–3757

Weber PC and Sellmayer A (1990) Modification of the prostanoid system and cell signalling by precursor fatty acids. *Adv Prostaglandin, Thrombox Leukotri Res* **21**: 217–224

*© 1999 Cancer Research Campaign*
